# Prediction of transmembrane helix orientation in polytopic membrane proteins

**DOI:** 10.1186/1472-6807-6-13

**Published:** 2006-06-22

**Authors:** Larisa Adamian, Jie Liang

**Affiliations:** 1 Department of Bioengineering, University of Illinois at Chicago, M/C 563, 835 S. Wolcott St, Chicago, IL 60612-7340, USA

## Abstract

**Background:**

Membrane proteins compose up to 30% of coding sequences within genomes. However, their structure determination is lagging behind compared with soluble proteins due to the experimental difficulties. Therefore, it is important to develop reliable computational methods to predict structures of membrane proteins.

**Results:**

We present a method for prediction of the TM helix orientation, which is an essential step in *ab initio *modeling of membrane proteins. Our method is based on a canonical model of the heptad repeat originally developed for coiled coils. We identify the helical surface patches that interface with lipid molecules at an accuracy of about 88% from the sequence information alone, using an empirical scoring function LIPS (LIPid-facing Surface), which combines lipophilicity and conservation of residues in the helix. We test and discuss results of prediction of helix-lipid interfaces on 162 transmembrane helices from 18 polytopic membrane proteins and present predicted orientations of TM helices in TRPV1 channel. We also apply our method to two structures of homologous cytochrome b_6_f complexes and find discrepancy in the assignment of TM helices from subunits PetG, PetN and PetL. The results of LIPS calculations and analysis of packing and H-bonding interactions support the helix assignment found in the cytochrome b_6_f structure from green alga but not the assignment of TM helices in the cyanobacterium b_6_f structure.

**Conclusion:**

LIPS calculations can be used for the prediction of helix orientation in *ab initio *modeling of polytopic membrane proteins. We also show with the example of two cytochrome b_6_f structures that our method can identify questionable helix assignments in membrane proteins. The LIPS server is available online at .

## Background

A significant increase in the number of structures of alpha helical membrane proteins in recent years revealed a remarkable complexity of interacting transmembrane (TM) helices. A great variation in length, shape, and tilt angles relative to the membrane plane is found in helical membrane proteins. For example, a structure of protein transporter (1RH5) contains a helix that is only about one half the length of the TM region, the structure of ClC chloride channel (1KPL) contains discontinuous helices, while all aquaporin structures (1FX8, 1J4N, 1RC2) contain two half helices important for function. Some TM helices are tilted and packed within the helical bundle so that they are only partially exposed to the membrane. Membrane proteins with ten or more TM helices may have helices that are completely buried within the helical bundle [1]. This great diversity only reflects the conformational space of integral alpha-helical membrane proteins sampled by the existing 30+ unique structures. If the number of structures of integral membrane proteins grows exponentially as predicted [2], even greater complexity in structural elements can be expected for transmembrane domains. This poses a great challenge for prediction and modeling of polytopic membrane proteins [3].

Recently, the structures of homo-oligomeric transmembrane (TM) proteins were successfully modeled using the techniques of simulated annealing, molecular dynamics [4-7], Monte-Carlo simulations [8], and an empirical scoring function designed to specifically distinguish tightly packed TM oligomers [9]. *Ab initio *modeling of structures of polytopic membrane proteins is more complicated [10]. Recently, the Rosetta structure prediction method, which uses a new membrane-specific version of the Rosetta low-resolution energy function, was successfully implemented for the prediction of structures of polytopic membrane proteins [11]. Other methods such as MembStruk [12] and PREDICT [13] start from ideal helices that are later subjected to "coarse" and "fine" optimization steps by energy minimization and molecular dynamics simulations. A required step in these methods is the prediction of TM helix orientation. Typically, a hydrophobic moment for every TM helix is determined [14] under the assumption that the hydrophobicity moment should point in the direction towards the lipid bilayer. However, Stevens and Arkin [15] showed that hydrophobicity moment alone is a poor indicator of the lipid-accessible surface in membrane proteins.

A strategy to improve the accuracy of prediction of helix orientation is to take advantage of available evolutionary information. It is well known that solvent-exposed residues in both soluble [16] and membrane [17-19] proteins are less conserved than buried residues. Komiya et al [20] proposed a method for characterizing the exposure of α-helices to the membrane that was based on the periodicity of conserved residues. Taylor et al [10] developed an automatic method that can proceed from a scan of the protein sequences to a predicted three-dimensional structure. In this method, finding non-conserved hydrophobic positions in multiple sequence alignments identifies the lipid-exposed surfaces of TM helices. The same idea was used to aid in the building of an alpha-carbon template for the TM helices of rhodopsin [21]. Briggs et al [22] suggested the use of widely available evolutionary information to find variable residues within homologous TM helices that are not important for the native structure. This information then can be used as constraints for global searching molecular dynamics simulations. Recently, an automated method for the analysis and prediction of buried and exposed residues of TM proteins with an impressive prediction accuracy of 80% was developed by Beuming and Weinstein [23]. This method is based on a new amino acid surface propensity (SP) scale derived from membrane protein structures and evolutionary conservation of buried and exposed residues. The probability of finding a residue in the protein interior is calculated for every residue in the TM helix. A cut-off value of this probability, which depends on the number of sequences in the multiple sequence alignment (MSA), is used to predict whether the residue is at an interior or exterior position.

In this study, we propose and extensively test a new approach for the prediction of helix-lipid interfaces of TM helices from sequence information alone based on a canonical model of the alpha helix. This method features a collective assessment of conservation and physico-chemical properties of the residues forming surface patches along the TM helix. Each surface patch is centered on one of the positions of the coiled coil heptad repeat and can be in contact with lipids or other helices. We score every patch with a scoring function, LIPS (LIPid-facing Surface), that combines a lipophilicity scale (TMLIP2) [24] and conservation entropy scores of residues [25]. We demonstrate using a set of 162 TM helices with helix-lipid interfaces that our method achieves an accuracy of 88%. We also describe how our method can be used to resolve inconsistency in experimental structures of cytochrome b_6_f complex orthologs.

## Results

### Heptad motif in TM helix-helix interactions

**Figure 1:**
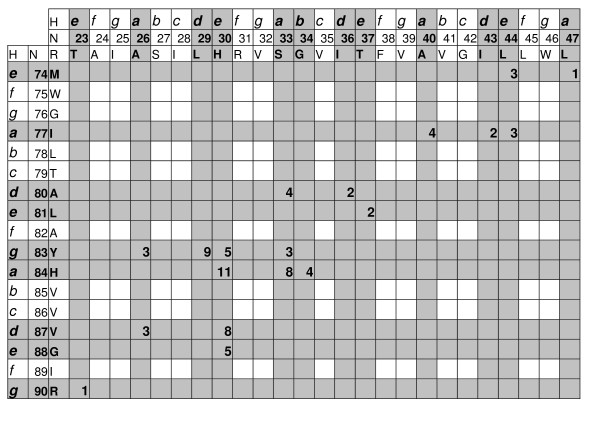
**Contact map of helix-helix interactions within the left-handed helical pair formed by helices tm I and tm II from SdhC subunit of *E. coli *succinate dehydrogenase (PDB: **1NEK). The three top rows and three left columns contain information on coiled coil heptad positions *abcdefg *(marked as **H**), residue numbers (**N**) and residue types (**R**) for tm I and tm II, respectively. Residues involved into interhelical interactions between these two TM helices are in bold. The number of atomic contacts as determined by INTERFACE calculations with probe radius of 0.5 Å between two interacting residues is listed at the intersections of the respective row and column. For example, Ser33 from tm I has 4 atomic contacts with Ala80, 3 atomic contacts with Tyr83, and 8 atomic contacts with His84 from tm II, while neighboring residue Val32 has no contacts with any residue from tm I.

The atomic interactions between two helices can be represented graphically as a two-dimensional atomic contact map as shown in Figure 1, which records the number count of the interhelical atomic contacts within the antiparallel left-handed helical pair formed by helices tm I and tm II of subunit SdhC of succinate dehydrogenase from *E. coli *(1NEK) [26]. We use the heptad motif as a structural template, where the structurally equivalent positions occur every two turns of the α-helix. The interacting residues on helices tm I and tm II of subunit SdhC can be mapped to the heptad repeat positions *a*, *d*, *e *and *g *by assigning the optimal starting position for the first interacting residue, *i.e*., when the first interacting residue of tm I (Thr23) is assigned to position *e *of the heptad repeat, the rest of the interacting residues can be assigned to positions *d*, *g*, *a *and *e *of the heptad repeat. Similarly, the correct starting point for mapping the interacting residue of tm II to the heptad repeat is position *e* for the first interacting residue Met74.

**Figure 2:**
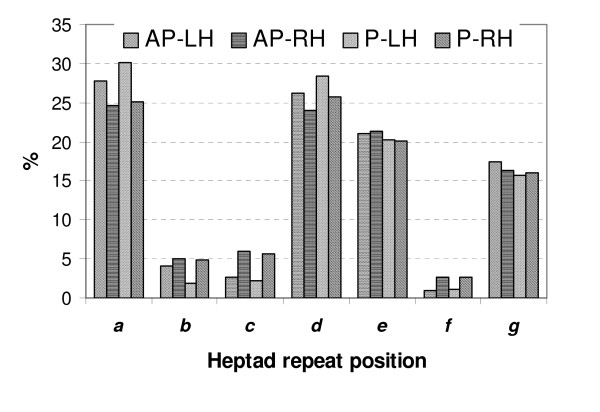
**Distribution of amino acid residues involved into interhelical interactions in transmembrane regions of membrane proteins at heptad repeat positions *abcdefg***. Types of helical pairs are: AP-LH – antiparallel left-handed; AP-RH – antiparallel right-handed; P-LH – parallel left-handed; P-RH – parallel right-handed. The combined frequencies of interacting residues from helix-helix interfaces at *a*, *d*, *e *and *g *positions in the heptad repeat are: left-handed parallel helical pairs, 94.7%; left-handed antiparallel helical pairs, 92.4%; right-handed parallel helical pairs, 86.9%; and right-handed antiparallel helical pairs, 86.5%.

We performed a similar mapping on 850 interacting helical interfaces from 425 TM helical pairs, where we obtained the best alignments of the interacting residues from every helical interface to the heptad repeat motif. Figure 2 summarizes the alignments of interacting residues in helices to the positions of the heptad repeat in 294 parallel (192 right-handed, 102 left-handed) and 546 antiparallel (222 right-handed, 324 left-handed) helical pairs. We find that the majority of interacting residues can be accurately mapped to heptad repeats.

Left-handed helical pairs have a higher fraction of interacting residues that can be mapped to canonical positions *a*, *d*, *e *and *g *of the heptad repeat (94.7% and 92.4% for parallel and anti-parallel helices, respectively). For right-handed parallel and antiparallel helical pairs, about 86.9% and 86.5% of interacting residues can be mapped to heptad repeats, respectively. In the right-handed helical coiled coils, structurally equivalent positions occur every three helical turns with the undecad repeat *a-k *[27,28]. Peters et al [27] found that the packing of residues in the right-handed coiled coils follows the general principle of knobs-into-holes packing of the left-handed coiled coils, with residues in the *a *and *h *positions of the undecad repeat structurally corresponding to residues in the *a *and *d *position of the heptad repeat, respectively. This allows us to use a heptad repeat as a simplified model for the interacting residues in the right-handed helical pairs as well.

### Helical faces

Since ~90% of all interacting residues in TM helices can be aligned to the heptad repeat positions, it is reasonable to assume that the residues forming lipid-accessible helical faces should follow similar patterns, as the degrees of freedom in the lipid-facing residues are constrained by the residues involved in helix-helix interactions, the majority of which are adequately characterized by the heptad motifs.

**Figure 3:**
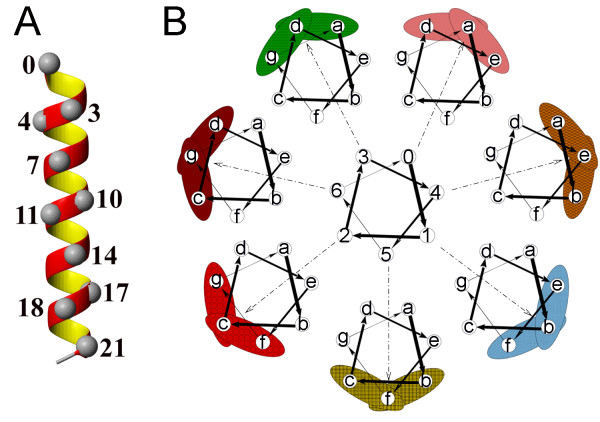
**Model of canonical helical faces**. (A) C_α _atoms that form a helical face are shown in space filling. Residues 3 and 4, 10 and 11 and 17 and 18 complement the anchoring residues 0, 7, 14 and 21, respectively. (B) Helical wheels illustrating the seven canonical helical faces centered at each of the seven positions of the heptad repeat. The central helical wheel contains numbers that label the helical faces 0 through 6. These numbers correspond to heptad positions *a *through *f *around which the helical surfaces are centered. The corresponding helical face is depicted as an outer wheel located next to that label. The outer helical wheels depict residues forming each of the seven helical faces, *e.g*., face 0 is formed by residues at heptad positions *a*-*d*-*e*, face 1: *b*-*e*-*f*, face 2: *c*-*f*-*g*, face 3: *d*-*g*-*a*, face 4: *e*-*a*-*b*, face 5: *f*-*b*-*c*, and face 6: *g*-*c*-*d*.

In the canonical model of the alpha helix [29], every seventh residue along the N- to C-terminus direction occupies a position that is roughly two turns away and underneath the previous one. The positions of every 7^th ^residue, "the anchoring residue", would form a slightly twisted surface along the helix, formed by 2 to 5 residues depending on the length of the helix. To make the number of residues in a TM helical face consistent, we add every third and fourth residues to the interfaces defined by the anchoring residues. These additional residues occupy positions one turn away from the anchoring residue. Figure 3A shows in spacefill the C_α _atoms that form such a helical face. Here, the helical face is centered around the anchoring residues "0", "7", "14", and "21" and complemented with residues "3" and "4", "10" and "11", and "17" and "18", respectively. Similarly, taking each of the seven positions of the heptad repeat in turn as the anchor position, we can build seven different helical faces. In Figure 3B, seven different helical wheels illustrate these seven faces anchored at each of the seven positions of the heptad repeat. The nodes of the central helical wheel contain numbers labeling the helical faces 0 through 6, which correspond to the heptad positions *a *through *f *as anchoring positions. The corresponding helical face is depicted by a helical wheel, located directly along the direction of the vector from the origin to the anchoring residue. The outer helical wheels depict residues forming each of the seven helical faces, *e.g*., face 0 is formed by residues at heptad positions *a*-*d*-*e*, face 1: *b*-*e*-*f*, face 2: *c*-*f*-*g*, face 3: *d*-*g*-*a*, face 4: *e*-*a*-*b*, face 5: *f*-*b*-*c *and face 6: *g*-*c*-*d*.

### Prediction of TM helix orientation

The orientation of a TM helix relative to a lipid bilayer can be predicted by finding either the mostly lipid-exposed or the mostly buried helical face. We explore both approaches by comparing prediction results for the mostly lipid-exposed helical faces, which should contain the least conserved but predominantly lipophilic residues, as well as the mostly buried helical faces, which should contain the most conserved and less lipophilic residues.

Table 1 summarizes the results of prediction of helix orientation for 162 helices from 18 membrane proteins for which a sufficient number of homologous sequences is available to compute positional entropy (see Methods section). The average lipophilicity of a helical face was calculated using TMLIP2 scale [24], which was recomputed every time to exclude the protein that was tested. TMLIP2 contains separate scales for the headgroup and hydrophobic regions of phospholipid bilayer to account for the different physico-chemical properties of the membrane. Table 1 lists the number of correctly predicted lipid-exposed and buried faces, based on the percentage of the lipid-accessible surface area of the residues forming a helical face. We compare prediction results when only the average helical face lipophilicity L¯f
 MathType@MTEF@5@5@+=feaafiart1ev1aaatCvAUfKttLearuWrP9MDH5MBPbIqV92AaeXatLxBI9gBaebbnrfifHhDYfgasaacH8akY=wiFfYdH8Gipec8Eeeu0xXdbba9frFj0=OqFfea0dXdd9vqai=hGuQ8kuc9pgc9s8qqaq=dirpe0xb9q8qiLsFr0=vr0=vr0dc8meaabaqaciaacaGaaeqabaqabeGadaaakeaacuWGmbatgaqeamaaBaaaleaacqWGMbGzaeqaaaaa@2F66@ was used, when only the average positional entropy E¯f
 MathType@MTEF@5@5@+=feaafiart1ev1aaatCvAUfKttLearuWrP9MDH5MBPbIqV92AaeXatLxBI9gBaebbnrfifHhDYfgasaacH8akY=wiFfYdH8Gipec8Eeeu0xXdbba9frFj0=OqFfea0dXdd9vqai=hGuQ8kuc9pgc9s8qqaq=dirpe0xb9q8qiLsFr0=vr0=vr0dc8meaabaqaciaacaGaaeqabaqabeGadaaakeaacuWGfbqrgaqeamaaBaaaleaacqWGMbGzaeqaaaaa@2F58@ of a helical face was used, and when the product of both lipophilicity and positional entropy (*S*_*f *_= E¯f
 MathType@MTEF@5@5@+=feaafiart1ev1aaatCvAUfKttLearuWrP9MDH5MBPbIqV92AaeXatLxBI9gBaebbnrfifHhDYfgasaacH8akY=wiFfYdH8Gipec8Eeeu0xXdbba9frFj0=OqFfea0dXdd9vqai=hGuQ8kuc9pgc9s8qqaq=dirpe0xb9q8qiLsFr0=vr0=vr0dc8meaabaqaciaacaGaaeqabaqabeGadaaakeaacuWGfbqrgaqeamaaBaaaleaacqWGMbGzaeqaaaaa@2F58@L¯f
 MathType@MTEF@5@5@+=feaafiart1ev1aaatCvAUfKttLearuWrP9MDH5MBPbIqV92AaeXatLxBI9gBaebbnrfifHhDYfgasaacH8akY=wiFfYdH8Gipec8Eeeu0xXdbba9frFj0=OqFfea0dXdd9vqai=hGuQ8kuc9pgc9s8qqaq=dirpe0xb9q8qiLsFr0=vr0=vr0dc8meaabaqaciaacaGaaeqabaqabeGadaaakeaacuWGmbatgaqeamaaBaaaleaacqWGMbGzaeqaaaaa@2F66@) was used.

**Table 1:** Results of prediction of buried and lipid-exposed faces of TM helices. The columns list the number of helices with correctly predicted lipid-exposed face or lipid-buried face when average lipophilicity (L¯f
 MathType@MTEF@5@5@+=feaafiart1ev1aaatCvAUfKttLearuWrP9MDH5MBPbIqV92AaeXatLxBI9gBaebbnrfifHhDYfgasaacH8akY=wiFfYdH8Gipec8Eeeu0xXdbba9frFj0=OqFfea0dXdd9vqai=hGuQ8kuc9pgc9s8qqaq=dirpe0xb9q8qiLsFr0=vr0=vr0dc8meaabaqaciaacaGaaeqabaqabeGadaaakeaacuWGmbatgaqeamaaBaaaleaacqWGMbGzaeqaaaaa@2F66@), average entropy (E¯f
 MathType@MTEF@5@5@+=feaafiart1ev1aaatCvAUfKttLearuWrP9MDH5MBPbIqV92AaeXatLxBI9gBaebbnrfifHhDYfgasaacH8akY=wiFfYdH8Gipec8Eeeu0xXdbba9frFj0=OqFfea0dXdd9vqai=hGuQ8kuc9pgc9s8qqaq=dirpe0xb9q8qiLsFr0=vr0=vr0dc8meaabaqaciaacaGaaeqabaqabeGadaaakeaacuWGfbqrgaqeamaaBaaaleaacqWGMbGzaeqaaaaa@2F58@) or LIPS function (*S *_*f*_) was used.

PDB	Helix count^1^	L¯f MathType@MTEF@5@5@+=feaafiart1ev1aaatCvAUfKttLearuWrP9MDH5MBPbIqV92AaeXatLxBI9gBaebbnrfifHhDYfgasaacH8akY=wiFfYdH8Gipec8Eeeu0xXdbba9frFj0=OqFfea0dXdd9vqai=hGuQ8kuc9pgc9s8qqaq=dirpe0xb9q8qiLsFr0=vr0=vr0dc8meaabaqaciaacaGaaeqabaqabeGadaaakeaacuWGmbatgaqeamaaBaaaleaacqWGMbGzaeqaaaaa@2F66@ Exp^2^	L¯f MathType@MTEF@5@5@+=feaafiart1ev1aaatCvAUfKttLearuWrP9MDH5MBPbIqV92AaeXatLxBI9gBaebbnrfifHhDYfgasaacH8akY=wiFfYdH8Gipec8Eeeu0xXdbba9frFj0=OqFfea0dXdd9vqai=hGuQ8kuc9pgc9s8qqaq=dirpe0xb9q8qiLsFr0=vr0=vr0dc8meaabaqaciaacaGaaeqabaqabeGadaaakeaacuWGmbatgaqeamaaBaaaleaacqWGMbGzaeqaaaaa@2F66@ Brd^3^	E¯f MathType@MTEF@5@5@+=feaafiart1ev1aaatCvAUfKttLearuWrP9MDH5MBPbIqV92AaeXatLxBI9gBaebbnrfifHhDYfgasaacH8akY=wiFfYdH8Gipec8Eeeu0xXdbba9frFj0=OqFfea0dXdd9vqai=hGuQ8kuc9pgc9s8qqaq=dirpe0xb9q8qiLsFr0=vr0=vr0dc8meaabaqaciaacaGaaeqabaqabeGadaaakeaacuWGfbqrgaqeamaaBaaaleaacqWGMbGzaeqaaaaa@2F58@ Exp^2^	E¯f MathType@MTEF@5@5@+=feaafiart1ev1aaatCvAUfKttLearuWrP9MDH5MBPbIqV92AaeXatLxBI9gBaebbnrfifHhDYfgasaacH8akY=wiFfYdH8Gipec8Eeeu0xXdbba9frFj0=OqFfea0dXdd9vqai=hGuQ8kuc9pgc9s8qqaq=dirpe0xb9q8qiLsFr0=vr0=vr0dc8meaabaqaciaacaGaaeqabaqabeGadaaakeaacuWGfbqrgaqeamaaBaaaleaacqWGMbGzaeqaaaaa@2F58@ Brd^3^	*S *_*f *_Exp^2^	*S *_*f *_Brd^3^
1C3W	7	2	2	7	7	7	6
1EUL	9	4	4	8	7	9	7
1FX8	4	1	0	3	3	3	3
1IWG	10	4	4	8	6	7	6
1J4N	4	4	2	3	4	4	4
1KPL	10	4	6	7	8	8	9
1KQF	5	4	2	4	3	5	3
1L9H	7	5	5	7	7	7	7
1M3X	11	7	6	9	6	10	8
1NEK	5	5	2	5	4	5	3
1OCR	24	14	11	18	13	21	11
1OKC	6	2	1	4	3	5	3
1PV6	12	6	3	9	4	9	6
1PW4	12	8	9	8	7	10	11
1Q16	5	3	5	5	3	5	5
1Q90	12	6	4	10	7	11	11
1RH5	9	6	2	7	3	7	3
1ZCD	10	5	3	9	6	10	8

Total	162 100%	90 56%	71 44%	131 81%	102 63%	143 88%	114 70%

^1 ^Number count of lipid-exposed TM helices.

^2 ^The lipid-exposed helical face is predicted correctly if the highest scoring face has the largest lipid accessible area.

^3 ^The buried helical face is predicted correctly if the lowest scoring face has the smallest lipid accessible area.

All lipid-exposed helical faces were determined correctly in 8 out of 18 proteins (Table 1). These proteins are relatively small and contain between 5 to 10 TM helices together with the strong ligand binding sites within the TM bundle, *e.g*., bacteriorhodopsin (1C3W) and rhodopsin (1L9H) contain covalently bound retinal, while succinate dehydrogenase (1NEK) and nitrate reductase A (1Q16) bind heme molecules. These binding sites provide strong evolutionary and physico-chemical constraints for the buried residues, resulting in strong discrimination between buried and exposed helical faces.

Results in Table 1 show that the prediction of the lipid-exposed faces is more reliable than the prediction of the buried faces. Indeed, we find that the face with the smallest lipophilicity index or the smallest entropy is not always the most buried: it often represents a tightly packed helix-helix interface and should not be used for prediction of helix orientation alone. There are 48 TM helices where prediction of buried faces has failed. We compare the positions of the predicted buried faces for these helices with the positions of the correct helical faces on the helical wheel (see Additional File 1). We find that in most cases the correct faces have significant overlap with the predicted faces. The anchor residues are shifted only by one residue on the helical wheel in 28 helices, and by two residues in 18 helices. For example, predicted buried face on TM4 (D) of bacteriorhodopsin is 6, while the correct face is 3. Helical wheel diagram (Figure 3B) shows that these two faces overlap by two residues and are neighbors to each other.

The best result (with average of 88% of correct predictions) was obtained for prediction of lipid-exposed helical faces with a scoring function that combines average lipophilicity and entropy. Using either lipophilicity or entropy scorings correctly predicts 55% and 81% of lipid-exposed helical faces, respectively. This result demonstrates that evolutionary conservation as measured by positional entropy provides stronger discrimination than lipophilicity. Prediction of the mostly buried helical face was less successful: the combined *S*_*f *_scoring function produced 70% of correct predictions, while lipophilicity and entropy scorings produced only 44% and 63% of correct predictions, respectively.

**Figure 4:**
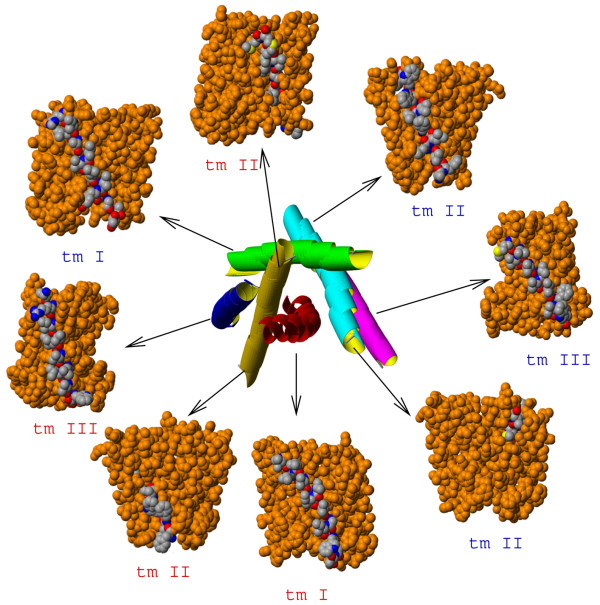
**Transmembrane region of succinate dehydrogenase (PDB:**1NEK**): top view of the helical bundle (center) and side views of the lipid accessible helices**. Predicted residues on the lipid accessible faces are shown in CPK color, the rest of the molecule is in orange. Labels for helices from the SdhC subunit are in red, and labels for helices from the SdhD subunit are in blue. Predicted lipid-facing residues from the tm II helices of SdhC and SdhD are shown on two separate side views, because these helices traverse the helical bundle and appear on two different sides of the TM domain.

TM helices may have very complex packing patterns within a helical bundle. For example, the tm II helices of SdhC and SdhD hydrophobic membrane anchor subunits of succinate dehydrogenase (SQR) cross the TM bundle with a large tilt angle and have lipid-exposed surfaces on the two opposite sides of the transmembrane domain. Our method works well for this complicated example. Figure 4 shows residues on the predicted lipid-exposed faces for all TM helices in SQR. There are two disconnected faces for both tm II helices from SdhC and SdhD subunits. The predicted lipid-exposed faces in each case contain two segments that are found to be lipid-accessible on opposite sides of the helical bundle.

### Prediction of lipid-exposed faces for structure-based and predicted TM helices

To assess how the definition of the TM helix boundaries affects the results of prediction of helix orientation, we compare the results of predicted helix orientations for structure-based and predicted TM helices in Leu transporter LeuT_Aa _from *Aquifex aeolicus *(PDB ID: 2A65), a bacterial homologue of Na^+^/Cl^- ^– dependent neurotransmitter transporter. All 12 TM helices are taken as predicted by the hidden Markov model topology predictor TMHMM [30]. With exception of helix 7, the sequences of computationally predicted and structure-based TM helices have significant overlap throughout the whole length of the helix (Table 2). There are two buried helices, which have most of their surfaces hidden within a helical bundle. These are not suitable for LIPS calculations, but can be predicted using RANTS scoring method of lipid accessibility [1]. The results of LIPS predictions of the lipid-exposed faces for the remaining 10 TM helices with TMHMM and structure-based sequences are summarized on Table 2. Correct predictions were made for both structure-based and computationally predicted TM helices for 5 helices with the N-terminal helix boundary difference ranging from 1 to 7 residues. For helix TM4, LIPS correctly predicted the lipid-exposed face for structure-based helix and gave the second best choice (with the majority of the predicted residues being lipid-accessible) for TMHMM helix. For helix TM9, LIPS correctly predicted the lipid-exposed face for TMHMM helix and failed for the structure-based helix. LIPS failed for both sequences of helix TM5, which differ only by 1 residue at the N-terminus and by 4 residues at the C-terminus. The second best choice was predicted for both structure-based and computed versions of helix TM12. LIPS correctly predicted lipid-exposed surface for structure-based TM7. We were unable to assess the correctness of the LIPS prediction for computationally predicted TM7 helix because the predicted sequence mainly represented the interhelical loop region according to 2A65 structure. The results on Table 2 demonstrate that predictions with identical outcome were obtained for 7 out of 9 helices for which the comparison between structure-based and computationally predicted sequences was plausible indicating a significant tolerance of the LIPS method to the definition of helical boundaries.

**Table 2:** Comparison of prediction of helix orientation for structure-based and computationally predicted helices in Leu transporter LeuT_Aa _from *Aquifex aeolicus *(PDB ID: 2A65).

**TM Helix**	**Source**	**First Residue Number**	**TM helix sequence**	**LIPS prediction**
1	Struct	15	**ILAMAGNAVGLGNF**LRFPVQ	Buried
	TMHMM	7	HWATRLGL**ILAMAGNAVGLGNF**	
2	Struct	41	**GAFMIPYIIAFLLVGIPLMW**	Correct
	TMHMM	39	GG**GAFMIPYIIAFLLVGIPLMW**I	Correct
3	Struct	92	**ILGVFGLWIPLVVAIYYVYI**ESWTLGFAIK	Correct
	TMHMM	89	FAK**ILGVFGLWIPLVVAIYYVYI**	Correct
4	Struct	166	**LFAYIVFLITMFINVSILI**	Correct
	TMHMM	165	S**LFAYIVFLITMFINVSILI**RGI	2^nd ^best
5	Struct	193	R**FAKIAMPTLFILAVFLVIR**	Wrong
	TMHMM	194	**FAKIAMPTLFILAVFLVIR**VFLL	Wrong
6	Struct	241	PG**VWIAAVGQIFFTLSLGFGAI**	Buried
	TMHMM	243	**VWIAAVGQIFFTLSLGFGAI**ITY	
7	Struct	279	GLTAATLNEKAEVI**LGGSI**	Correct
	TMHMM	293	**LGGSI**SIPAAVAFFGVANAVAIA	*
8	Struct	342	**FLWFFLLFFAGLTSSI**AIMQPMI	Correct
	TMHMM	335	AGGTFLG**FLWFFLLFFAGLTSSI**	Correct
9	Struct	379	**VLWTAAIVFFSAHLVMF**L	Wrong
	TMHMM	378	A**VLWTAAIVFFSAHLVMF**	Correct
10	Struct	398	KSLDEMD**FWAGTIGVVFFGLTELI**	Correct
	TMHMM	405	**FWAGTIGVVFFGLTELI**IFFWIF	Correct
11	Struct	450	**YVMRYITPAFLAVLLVVWAR**EYI	Correct
	TMHMM	447	IYY**YVMRYITPAFLAVLLVVWAR**	Correct
12	Struct	482	TV**WITRFYIIGLFLFLTF**	2^nd ^best
	TMHMM	484	**WITRFYIIGLFLFLTF**LVFL	2^nd ^best

* Predicted and structure-based TM helices have little overlap.

### Prediction of orientation of TM helices in transient receptor potential vanillin subtype 1 (TRPV1) channel

Members of the protein family of transient receptor potential (TRP) ion channels mediate a wide range of sensory responses, including thermosensation and taste. Vanillin receptor TRPV1, which possesses weak voltage sensitivity, is activated by warm temperatures and is a molecular sensor for detecting multiple pain-producing stimuli. TRPV1 is a key element for inflammatory nociception and an attractive drug target.

Although there are no high-resolution structures of TRP channels available at the present time, the overall structural organization of TRP channels should be similar to that of voltage-gated potassium (Kv) channels [31], which are composed of tetramers of subunits containing two pore-forming helices (S5 and S6) connected by a membrane-re-entrant loop P and the voltage-sensing transmembrane segments S1 to S4. There are several structures of voltage-dependent channels available, including the X-ray structure of a rat brain voltage-dependent Shaker family K^+ ^channel (PDB:2A79) [32]. TRPV1 has no significant sequence similarity with these sequences, indicating that it would be difficult to produce a reliable sequence alignment to build a homology model. Here, we use LIPS scoring to predict orientation of TM helices in TRPV1 channel.

**Figure 5:**
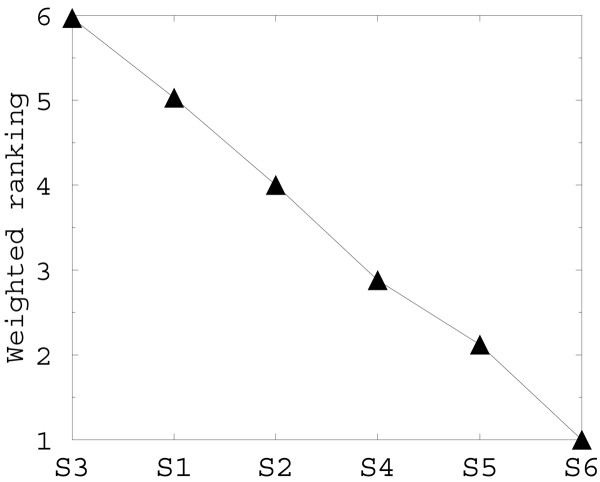
**Results of prediction of lipid-accessibility of TRPV1 TM helices S1–S6 by RANTS server [1]**. Ranking by solvent accessibility shows that TM helices S6 and S5 are the least lipid accessible, while helix S3 is the most lipid-accessible.

First, we predict the locations of the six TM helices of human TRPV1 channel (accession number CAB95729) using a hidden Markov model topology predictor TMHMM [30]. We then build a multiple sequence alignment profile for every TM helix and rank helices by their lipid accessibility using RANTS server [1]. Our ranking by lipid accessibility shows that TM helices S6 and S5 are the least lipid accessible, while helix S3 is the most lipid-accessible (Figure 5). This agrees well with the hypothesis that the transmembrane part of TRPV1 channel should be similar to that of voltage-gated K^+ ^channels.

**Figure 6:**
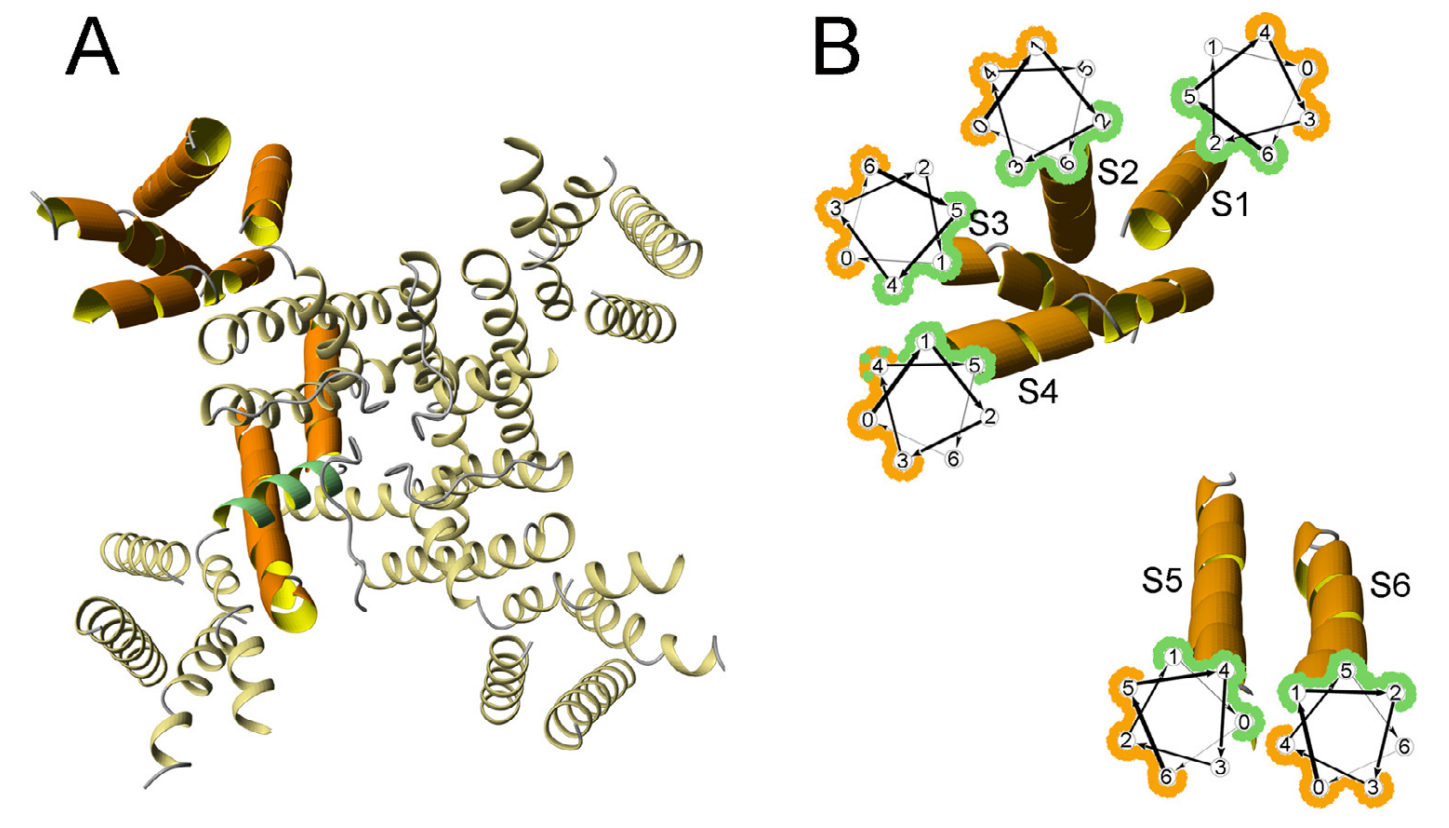
**Prediction of lipid-exposed and buried faces in TRPV1 TM helices**. (A) TM region of the rat brain K^+ ^channel (PDB: 2A79), which is used here as a structural model for TRPV1 channel. A monomer is shown in thick orange-and-yellow ribbons, while a helical part of the re-entrant loop is shown in green. B) K^+ ^channel monomer with helical wheel cartoons showing predicted lipid-exposed (outlined with orange) and buried (outlined with green) faces of TRPV1 channel. The residues forming buried and exposed faces are listed on Table 3.

Finally, we use multiple sequence alignment profiles for all TRPV1 TM helices to calculate LIPS scores for helical faces on every helix. The results of LIPS calculations are presented on Table 3, which gives information about helical faces with the largest and the smallest LIPS scores on helices S1–S6, as well as the residues found on the respective helical face. Figure 6 further illustrates the results of LIPS calculations by showing the TM region of the tetrameric rat brain K^+ ^channel structure (2A79, Figure 6A), which is used here as a structural template for TRPV1 channel, and the structure of the TM region of a single monomer with cartoons of helical wheels with predicted lipid-exposed and buried faces highlighted by orange and green lines, respectively (Figure 6B). Figure 6B also shows that the exposed and buried faces are predicted on the opposite surfaces for all TM helices with the exception of helix S4, where the buried and exposed faces overlap, which may indicate that the prediction for this helix is incorrect.

**Table 3:** Summary of prediction of lipid-facing and buried residues in transmembrane region of TRPV1 channel

**TM Helix**		**Helical Face**	**LIPS score**	**Helical face residues**
**S1**	Buried	2	4.65	K432 F435 Y436 F439 Y442 C443 M446 F449 T450 A453 R456
	Exposed	0	9.35	F430 R433 I434 F437 L440 V441 L444 I447 I448 M451 Y454 Y455
**S2**	Buried	6	3.55	R474 G477 E478 S481 G484 G485 F488 R491 G492 Y495 G470 D471
	Exposed	4	8.50	Y472 V475 T476 I479 V482 L483 V486 F489 F490 I493 K468 T469
**S3**	Buried	1	5.11	S512 L515 F516 Q519 F522 M523 T526 L529 Y530 H533
	Exposed	3	9.53	M514 F517 L518 L521 L524 A525 V528 F531 S532 K535
**S4**	Buried	1	2.96	Y537 S540 M541 S544 L547 G548 N551 Y554 Y555 G558
	Exposed	0	5.61	E536 A539 S540 F543 A546 L547 T550 L553 Y554 R557 Q560
**S5**	Buried	4	3.23	F580 V583 Y584 F587 G590 F591 A594 T597 L598 D576 L577
	Exposed	2	6.68	C578 M581 F582 V585 L588 F589 S592 V595 V596 I599
**S6**	Buried	5	3.01	F660 L663 L664 Y667 L670 T671 L674 N677 M678 A681 G684 E685 F656 K657
	Exposed	0	5.44	D655 A658 V659 I662 L665 A666 I669 Y672 I673 L676 L679 I680 M683 T686

### Assessment of different structural assignment in homologous membrane proteins: two cytochrome b_6_f structures (1Q90 *vs*. 1VF5)

**Figure 7:**
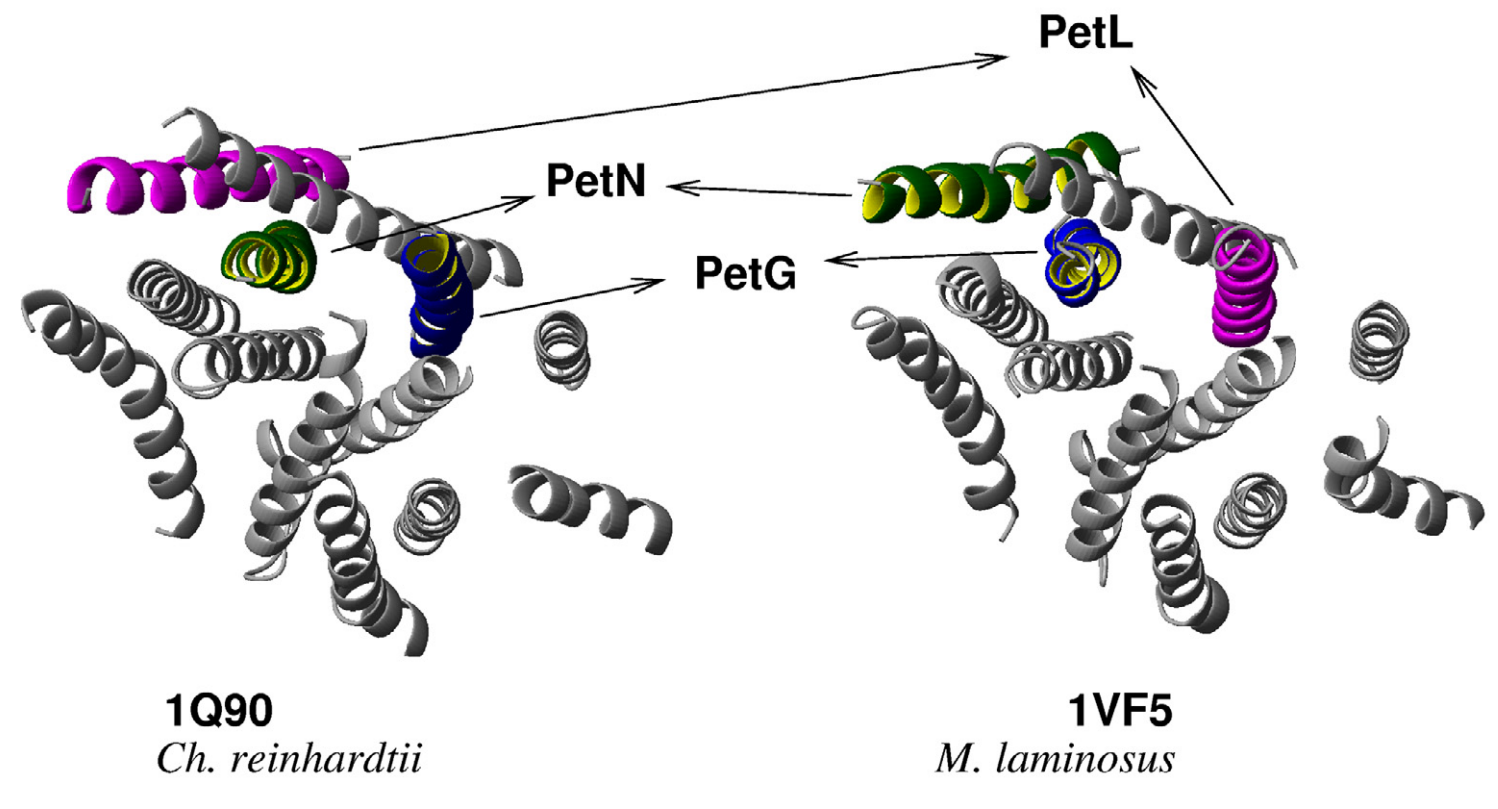
**Transmembrane domains of cytochrome b_6_f complexes from *Ch. reinhardtii *(PDB:**1Q90**) and *M. laminosus *(PDB: **1VF5**)**. PetG subunit (blue-and-yellow helix), PetL subunit (magenta) and PetN subunit (green-and-yellow) in 1Q90 and 1VF5 structures. The overall positioning of TM helices is very similar in both structures, while the assignment of subunits PetG, PetL, and PetN is different.

The multisubunit cytochrome b_6_f complex mediates electron transfer between the photosystem II and the photosystem I reaction centers, in which H_2_O is the electron donor [33]. Each cytochrome b_6_f monomer contains eight subunits and seven natural prosthetic groups. Almost identical structures of cytochrome b_6_f complex were obtained from the thermophilic cyanobacterium *Mastigocladus laminosus *(PDB: 1VF5) [34] and the green alga *Chlamydomonas reinhardtii *(PDB: 1Q90) [35]. Six out of eight polypeptide chains with TM helices are highly homologous in both structures, with sequence identity between the respective orthologous chains of 56.6% for cytochrome f, 83.7% for cytochrome b_6_, 78.1% for subunit IV, 57.9% for PetG, 45.2% for PetN, and 37.5% for Rieske iron-sulfur protein. Two small polypeptides, PetL and PetM, have a low sequence identity of 26.5% and 25.5% and contain only one TM helix. The overall positioning of TM helices is very similar in both structures (Figure 7), while the assignment of subunits PetG, PetL, and PetN relative to each other is different – a phenomenon, which may be accounted for by evolution over the 10^9 ^years [33] that separated the two species. Alternatively, it could be the result of different interpretations of the electronic density maps of low resolution (3.0 Å and 3.1 Å for 1VF5 and 1Q90, respectively). We examined the pattern of packing and conservation of residues in the TM helices of subunits PetG, PetL and PetN from both structures. Our overall findings suggest that the placement of the subunits in cyanobacterial structure 1VF5 should follow the same pattern as that of green alga structure 1Q90. Our analysis is described in more details below and supplemented by multiple sequence alignments for TM helices of subunits PetG, PetL, PetN, and PetM (see Additional File 2).

#### Packing of PetN subunit with PetL and PetM subunits

**Figure 8:**
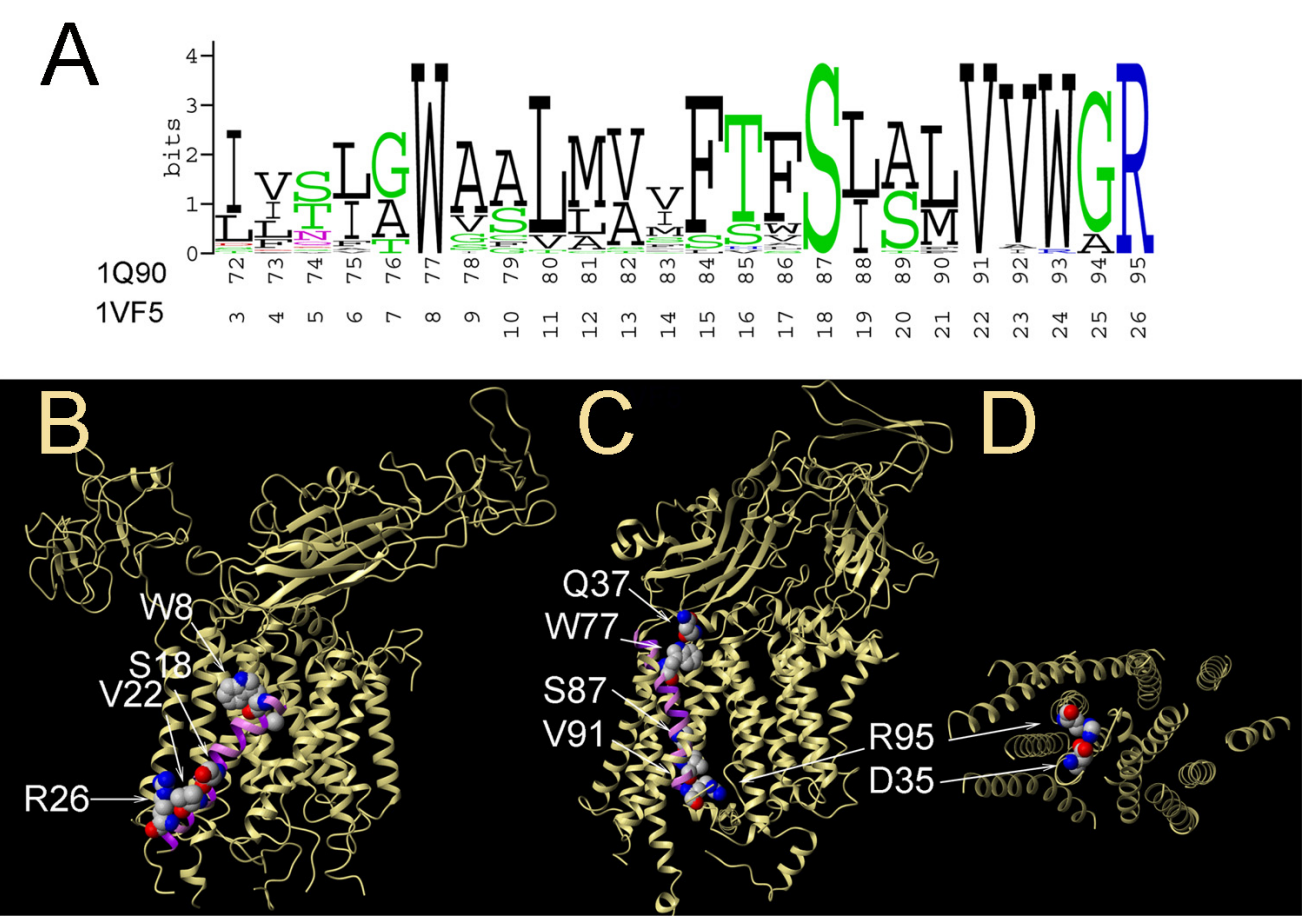
**Conservation of residues and lipid-exposed surfaces in PetN subunit of cytochrome b_6_f**. (A) Sequence logo for PetN subunit (31 sequences, pairwise sequence identity 29%–88%). (B) Highly conserved residues Trp8, Ser18, Val22, and Arg26 face phospholipids in 1VF5 structure. (C) Highly conserved residues Ser87 and Val91 are buried within the helical bundle in 1Q90 structure. Residue Trp77 forms a hydrogen bond with conserved Gln37 from the soluble domain of cytochrome f. (D) Interhelical intersubunit hydrogen bond between Arg95 from PetN and Asp35 from subunit IV in 1Q90 (only TM domain is shown for clarity).

Placement of the PetN TM helix is different in 1Q90 and 1VF5 structures: it is almost completely buried within a helical bundle in 1Q90 and is exposed in 1VF5 (Figure 7). Blast searches with the PetN sequences of green alga and cyanobacterium against a non-redundant database yielded 31 sequences with pairwise sequence identity between 29%–88%. LIPS calculations correctly predicted lipid-accessible face containing residues Ile72, Ile75, Gly76, Ala79, Val82, Met83, Phe86, Ser89, Leu90, and Trp93 in the case of 1Q90. Of these residues, only Ile72, Ile75, and Met83 are accessible to the probe with radius 1.9 Å. These probe-accessible positions are among the least conserved in the MSA of the PetN subunit in the TM region as seen on the sequence logo (Figure 8A). The analogous face in 1VF5 also receives a high LIPS score and partially faces phospholipids. However, this surface is not the most lipid-exposed in the cyanobacterium cytochrome b_6_f complex. In fact, the highest lipid accessibility is observed for two PetN helical faces from 1VF5, which contain highly (100%) conserved Trp8, Ser18, Val22, and Arg26 (Figure 8B), and have lower LIPS scores. The analogous faces with conserved residues in the PetN TM helix from 1Q90 are buried within a helical bundle and are mainly involved in helix-helix interactions with helix I from subunit IV (Figure 8C). Remarkably, there is an interhelical intersubunit H-bond between highly conserved (100%) Arg95 (PetN) and Asp35 (subunit IV) in 1Q90 (Figure 8D). However, Arg26 in PetN from cyanobacterium, a counterpart of Arg 95 in green alga, faces lipids with its side chain aligned along the TM helix and points into the middle of phospholipid bilayer (Figure 8B). Such anti-snorkeling behavior is not characteristic for the Arg side chain in this region of the membrane as shown by Granseth et al [36] and reviewed by Liang et al [37].

Another highly conserved residue in the PetN subunit is Trp77/Trp8 in 1Q90 and 1VF5, respectively. In the cyanobacterium structure (1VF5), Trp8 points towards the lipid phase (Figure 8B), while in the green alga structure (1Q90) this residue points inside of TM bundle into the lipid-filled cavity and forms a H-bond with highly conserved Gln37 from the soluble domain of cytochrome f (Figure 8C). Both positions of Trp residues are feasible, although the H-bond with highly conserved Gln37 from cytochrome f in green alga adds to the support of the helix packing pattern in 1Q90 structure. Additionally, Trp8 in 1VF5 has a highly unfavorable dihedral phi angle of -132°, with the ideal value being around -65°. Trp77 in 1Q90 has a more favorable phi angle of -54.2° (Dihedral angle data are obtained from PDB).

**Figure 9:**
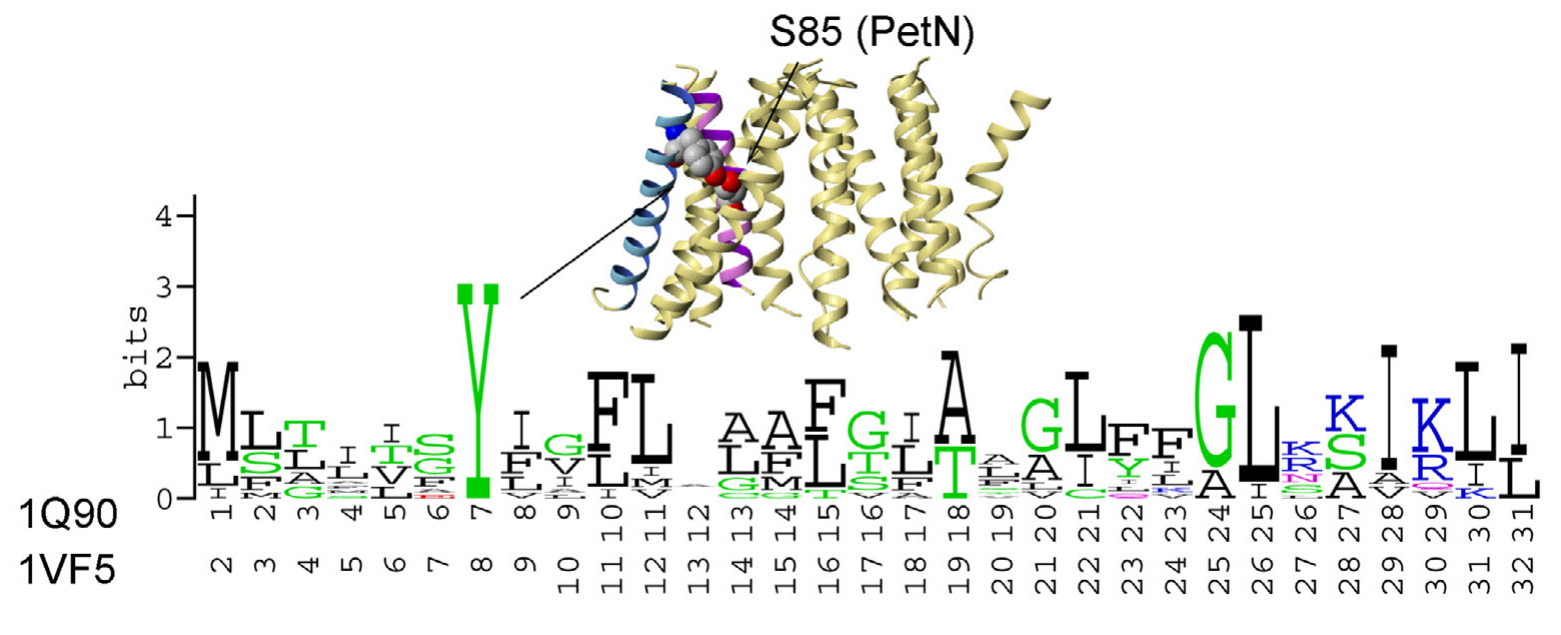
**Conservation of residues in PetL subunit of cytochrome b_6_f**. Sequence logo for PetL subunit (11 sequences, pairwise sequence identity 13%–87%). Conserved residues Tyr7 from PetL and Ser85 from PetN form a possible interhelical hydrogen bond in 1Q90 (shown in spacefill).

The N-terminal end of the PetN TM helix interacts with the TM helix of PetL subunit in the green alga structure 1Q90, and with PetG subunit in 1VF5. A BLAST search of the non-redundant database with the PetL sequences from green alga and cyanobacterium yielded 11 sequences with pairwise sequence identity between 13% – 87%. LIPS calculation correctly predicted the lipid-accessible and inaccessible faces in 1Q90, while it failed again in 1VF5. The sequence logo on Figure 9 shows that the PetL subunit has overall high sequence variability with only one 100% conserved residue (Tyr7 in 1Q90 and Tyr8 in 1VF5). A possible interhelical H-bond between the hydroxyl groups of Tyr7 and Ser85 in 1Q90 was detected by HBPLUS [38] (Figure 9). Although position 85 in PetN is rather variable, the sequence logo of the PetN subunit (Figure 8A) shows that the homologous sequences mainly contain Ser or Thr residues, both of which can form a H-bond with hydroxyl group of Tyr.

**Figure 10:**
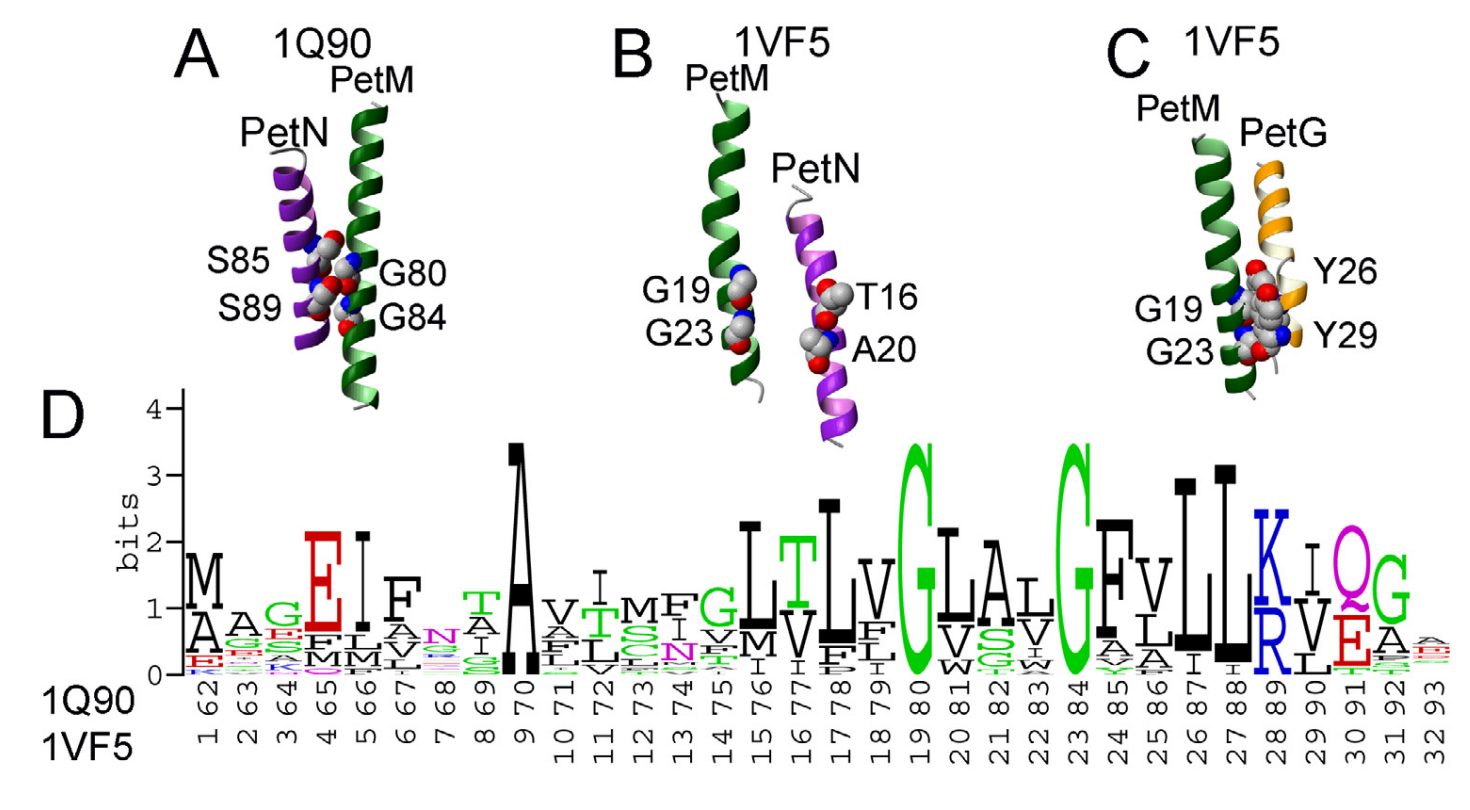
**Conservation of residues and packing interactions of PetM subunit in cytochrome b_6_f**. (A) TM helices PetN and PetM are tightly packed around the overrepresented sequential motifs SS4 and GG4 in 1Q90. Small residues such as Gly and Ser promote tight packing and form weak hydrogen bonds [40] at helix-helix interfaces. (B) TM helices PetN and PetM do not pack tightly in 1VF5 structure. (C) Conserved GG4 motif on the PetM helix packs against Tyr26 and Tyr29 residues on the PetG helix in 1VF5. (D) Sequence logo of PetM subunt (17 sequences, pairwise sequence identity 22%–81%).

Residues Ser85 and Ser89 form an SS4 motif (following the nomenclature of Senes et al [39]) on the PetN TM helix, and pack tightly against Gly80 and Gly84 (GG4 motif [39]) on the subunit PetM in 1Q90, as shown on Figure 10A. Interaction of small residues is usually characteristic of tight van der Waals packing and weak hydrogen bonds [40], which provide additional stability and specificity to interhelical interactions. Analysis of tightly packed interhelical triplets of amino acid residues showed that SS4 and GG4 sequence motifs are often found at the interhelical crossings of helices in polytopic integral membrane proteins [41]. Figure 10B demonstrantes that subunits PetN and PetM from the cyanobacterium structure do not form any tight interhelical interactions. Here, the conserved GG4 motif on the subunit PetM is found packed against Tyr26 and Tyr29 from the subunit PetG (Figure 10C). The homologue of the SS4 motif in PetN from green alga is TA4 motif in cyanobacterium, which is composed of Thr 16 and Ala 20. No tight packing is found around these two residues in 1VF5.

#### Packing of the PetG and PetM subunits

**Figure 11:**
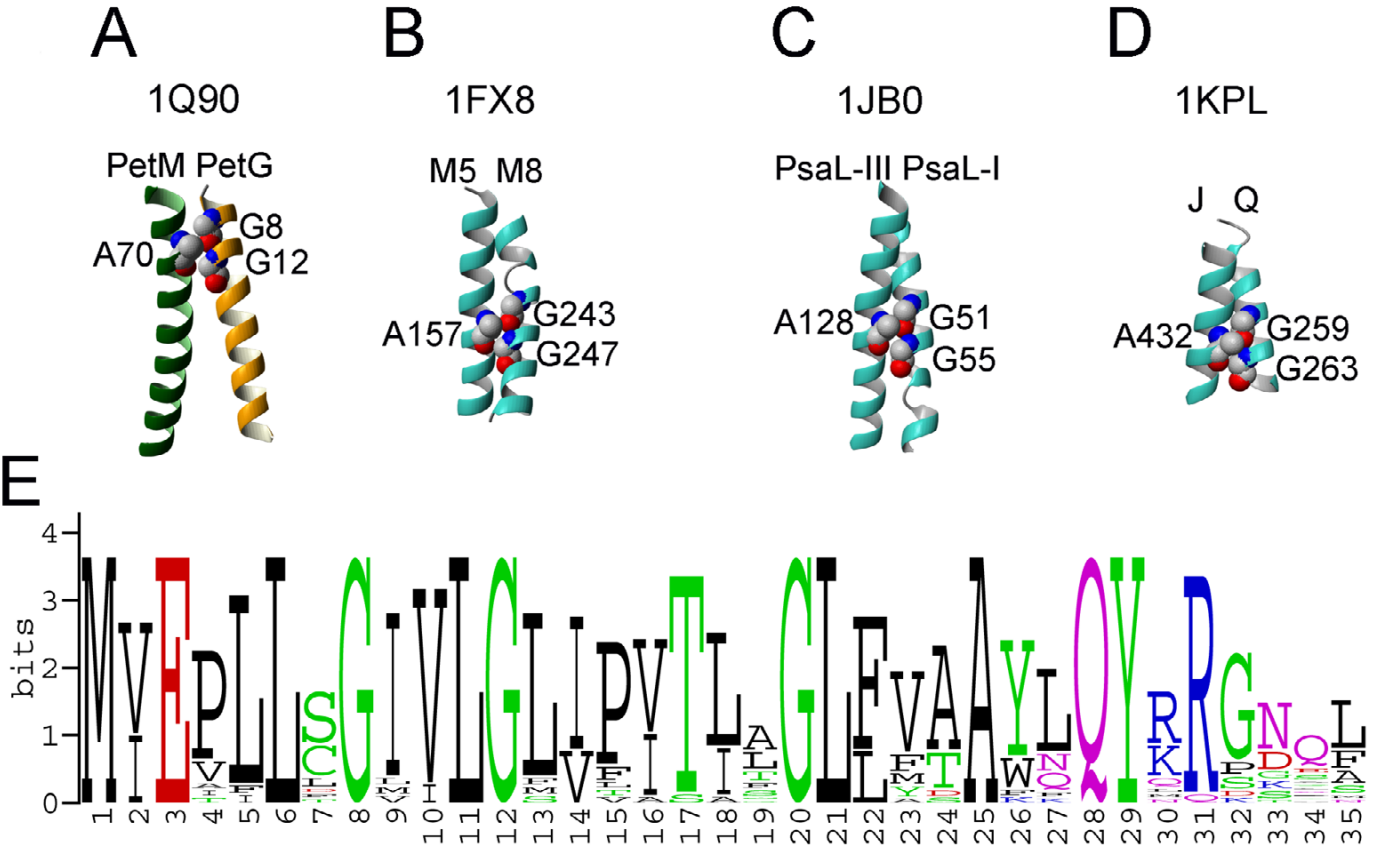
**Packing of PetG and PetM subunits in cytochrome b_6_f complex**. (A) AGG triplet is formed by highly conserved residues Gly8 and Gly12 from PetG and Ala70 from PetM in 1Q90 structure. (B-D) Structurally similar AGG triplets are found in glycerol facilitator (1FX8), photosystem I (1JB0), and ClC Cl^- ^chloride channel (1KPL). (E) Sequence logo of the PetG subunit (21 sequences, pairwise sequence identity 46%–89%).

The TM helices of the PetG and PetM subunits form a right-handed parallel helical pair with a crossing angle of -44° in 1Q90 (Figure 11A). The helical crossing point occurs around two highly conserved residues (Gly8 and Gly12) forming a GG4 motif (see the sequence logo on Figure 11E) on the PetG subunit, and highly conserved Ala70 (Ala9 in 1VF5) from the PetM subunit (Figure 10D) These residues form a tightly packed high propensity AGG residue triplet (Figure 11A) [41]. AGG triplets with GG4 motif on one helix and Ala residue on the other helix were found in several other membrane proteins, such as glycerol facilitator (1FX8, Figure 11A), photosystem I (1JB0, Figure 11C), and ClC Cl^- ^chloride channel (1KPL, Figure 11D). Remarkably, all these triplets have similar configurations of residues and occur on parallel helical pairs with crossing angles in the -33° – -48° range [41] implying that this conserved structural motif is often used in membrane proteins to promote folding and stability of membrane proteins. The respective GG4 sequential pair is present in the cyanobacterium PetG sequence as well; however, it does not participate in any interhelical interaction. In fact, only one of the conserved glycine residues (Gly12) is resolved in 1VF5, while Gly8 is out of the TM region.

In summary, we find that the conserved faces of PetN, PetL, and PetG subunits are hidden within the helical bundle in green alga structure 1Q90, while in cyanobacterium structure 1VF5, the conserved helical faces of the same subunits are often lipid-exposed. In addition, H-bonding potentials of amino acid side chains are better satisfied in the structure of green algae than in the cyanobacterium structure. Sequence motifs such as GG4 and SS4 in PetG, PetL and PetN subunits are found in tight helix-helix packing interactions in the green alga cytochrome b_6_f structure, while such tight packing interactions are often missing in cyanobacterium 1VF5 structure. Although our analysis is only suggestive, it shows that our method can be helpful in resolving discrepancy in the assignment of helical packing of low-resolution structures of membrane proteins.

## Discussion

The LIPS method uses TMLIP lipophilicity scales [24] and conservation of the residues in the TM helix. A similar approach (ProperTM) was used by Beuming and Weinstein for prediction of lipid-facing residues in membrane proteins [23]. The difference between these two methods is that LIPS is based on an explicit surface models and the collective assessment of the physico-chemical and conservation properties of the residues forming a patch on the surface of the TM helix. In the original Beuming and Weinstein method [23], a cut-off value is used to classify a particular residue as buried or exposed. The model of helical faces was not used. Here, each of the seven overlapping patches is scored and ranked, eliminating the need for the sequence-dependent cut-off to classify a residue as buried or lipid-accessible. Another difference is in the calculation of lipid propensity. In TMLIP2, lipid propensity is normalized to remove the bias of residue composition. In [23], the calculation of Surface Propensity values for all residues are scaled, so they fall between 0 and 1, but are not normalized by residue composition.

The construction of helical patches is based on the structural analysis of helix-helix interfaces in 29 multispan membrane proteins, which showed the abundance of interacting heptad motifs in the TM helix-helix interfaces. Our data are in agreement with results obtained by Langosh and Heringa [42] using a nearest neighbour analysis on a set of three membrane proteins. They showed that 97% of all residues identified within the left-handed TM helix-helix interfaces conformed to the heptad repeat pattern found in soluble coiled coils. However, it should be noted that the packing of TM helices in membrane proteins is much more irregular than that found in the soluble coiled coils, where the concept of heptad repeats originated. We use the heptad repeat here only to refer to the interacting interfaces, and do not suggest that the overall global organization of TM helices is coiled coils.

Although the overall accuracy of prediction of lipid-exposed helical faces in TM helices is almost 90%, the LIPS calculations failed to correctly predict lipid-exposed faces in 19 out of 162 lipid-accessible helices. Table 4 lists some of these helices and summarizes possible reasons why our predictions did not succeed for these proteins. Among the 8 cases listed, we find that three are due to the specific binding of cardiolipin, two are due to irregular helix breaking, and one case is due to the overwhelming amount of protein-heme interaction. Heme binding was not explicitly modelled in this study because we only consider interhelical and helix-lipid interactions, and, therefore our prediction failed for this protein.

**Table 4:** Summary of TM helices for which LIPS failed to correctly predict the lipid-exposed surfaces. Rationalization of the failure is listed for some helices.

**Protein**	**Helix**	**Rationalization**
1IWG	TM2	(N/C)* LIPS offers a second best choice of lipid-exposed helical face
	TM8	Regular helical structure is broken around A889 and A890. LIPS offers a second best choice
1KQF	TM1	Cardiolipin binding
1M3X	TM11	Cardiolipin binding
1OCR	TM18	(N/C) LIPS offers a second best choice
1PV6	TM4	Regular helical structure is broken around N119 by P123
1PW4	TM12	(N/C) LIPS offers a second best choice
1Q90	TM2	Heme interaction

* N/C: Not clear.

The failure to predict surfaces interacting with cardiolipin is expected because of the high specificity of cardiolipin binding. A previous evolutionary analysis of sequences of reaction centers showed that the cardiolipin interaction sites are preserved in terms of size, shape, and charge distribution by retaining certain types of amino acid side chains at positions interacting with cardiolipin over a wide range of photosynthetic bacteria [43]. This affects the conservation component of the LIPS function by reducing the average entropy of the lipid-exposed face and resulting in a prediction that the involved residues are not lipid-exposed.

**Figure 12:**
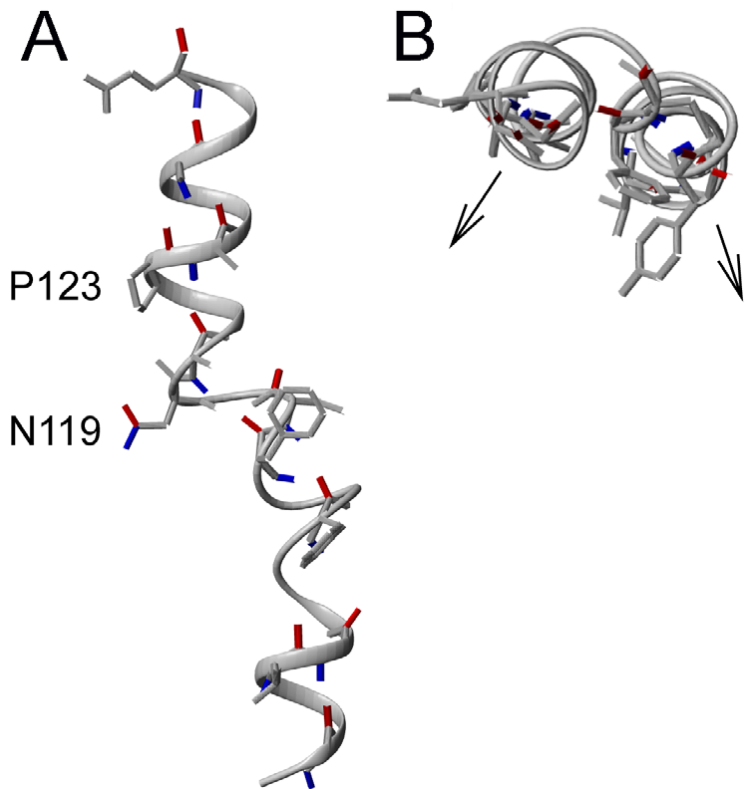
**Example of a swivel-type kink**. A swivel-type kink in proline-containing (P123) TM4 helix of lactose permease (PDB:1PV6), where N- and C-terminal halves of the helical face with the largest probe-accessible area are rotated by ~80 at residue Asn119 relative to the axis connecting the N- and C-termini of the helix. (A) Side view. (B) Arrows show that orientation of the mostly lipid-accessible helical face is different in the N-terminaland C-terminal regions of the helix.

Our analysis of failed predictions also shows that the seven canonical helical faces method, which is based on a heptad repeat motif, may not be applicable to helices with kinks formed around Pro or Gly residues. Analysis of proline-containing TM helices showed that proline residues are often found in the central region of TM helices, where they may induce hinges that can be described by a kink angle θ and swivel angle τ [44]. We find that the outcome of the prediction of helix orientation for proline-containing helices using a canonical helical face approach is rarely affected by the proline-induced kinks, if the swivel angles are small. For example, residues involved in interhelical interactions in the proline-containing helices B, C and F of bacteriorhodopsin can be described by the continuous sequence of *a*, *d*, *e *and *g *registers without any interruption, which results in the correct prediction of the lipid-exposed helical face. The large swivel angles at Pro-induced kinks result in rotation of one part of the helix relative to another. In such cases, residues forming a helical face may end up on two different sides of the helix after passing the swivel point, as seen in the proline-containing (Pro123) TM4 helix of lactose permease (1PV6), where the N- and C-terminal halves of the helical face with the largest probe-accessible area are shifted by ~80° relative to the helical axis at residue Asn119 (Figure 12). Fortunately, this type of kink does not occur frequently. Overall, our analysis of the interaction patterns in the TM helix-helix interfaces showed that the helix kinks induced by proline residues rarely interrupt the heptad motif for contact interfaces.

There are 11 failed predictions out of the 19 where we do not have adequate rationalization. In three out of these 11 cases, LIPS proposed the second best choice for the lipid-exposed face that shares a substantial number of residues with the correct helical face. Because different membrane proteins have homologous sequences from different species, the computed entropy values are not directly comparable and the absolute value of LIPS score cannot be used as a quality indicator. To increase the probability of the correct prediction, one should obtain as many orthologous sequences of the protein of interest as possible. It is also useful to analyse helical faces with the largest and the smallest LIPS scores together as we have shown on the example of TRPV1 channel: the assignment of lipid-facing and buried surfaces on the opposite sides of the helix is often an indicator of a correct prediction. In addition, if the boundaries of the helix are not known, it would be very helpful to run several calculations to find the residues of the high and low scoring faces and to derive a consensus lipid-exposed or buried face.

Finally, we have excluded TM helices that occur at the physiologically important protein-protein interfaces from the dataset for prediction of TM helix-lipid interfaces for two reasons. First, because the residues at the physiologically important protein-protein interfaces of the membrane proteins are more conserved than the lipid-facing residues and less conserved than the core residues [18], the modeling of the evolution of TM helices at the protein-protein interfaces would require a more elaborate approach. Second, a propensity scale derived from the residues occurring at the protein-protein interfaces should be applied instead of TMLIP lipophilicity scales, which were derived from the oligomeric structures of the membrane proteins and are not applicable to the residues at protein-protein interfaces.

A limitation of the current method is that the entropy calculation does not explicitly takes into account the phylogenetic relationships among the homologous proteins. A future direction would be to explore the possibility of using more advanced estimates of the evolutionary conservation, such as Evolutionary Trace [45], REVCOM [46], CONSURF [47], and other available methods [48,49]. Another direction is to study the compatibility of proposed TM helix assignment directly with electron density maps.

## Conclusion

In this study, we have developed a method (LIPS) for prediction of the orientation of TM helices by locating lipid-protein interfaces. LIPS is based on the canonical helical face model derived from the heptad repeat motif, which allows collective assessment of the evolutionary and physico-chemical properties for each of the seven faces formed by residues centered at one of the seven positions of the heptad repeat. The LIPS scoring function predicts orientation of lipid-exposed TM helices in polytopic membrane proteins with an accuracy of 88%. Our method can be used for the prediction of helix orientation in the *ab initio *modeling of polytopic membrane proteins. We also show that LIPS calculations can identify questionable helix assignments in membrane proteins with the example of two cytochrome b_6_f structures.

## Methods

### Computation of interhelical atomic contacts

Computation of interhelical atomic contacts on a set of 29 transmembrane proteins was performed using the alpha shape application program INTERFACE [50-52] as described previously [53]. The PDB names for the protein structures used: 1C3W, 1E12, 1EHK, 1EUL, 1FX8, 1H2S, 1IWG, 1J4N, 1JB0, 1K4C, 1KB9, 1KF6, 1KPL, 1KQF, 1L7V, 1L9H, 1M3X, 1M56, 1MSL, 1NEK, 1OCR, 1OKC, 1PP9, 1PV6, 1PW4, 1Q16, 1QLA, 1Q90, 1RH5. To estimate the pattern of heptad repeat positions involved in helix-helix interactions, we used helical pairs with at least three interacting residues. Amino acid residues from every helix in a helical pair involved in the interhelical interaction form a helical face, regardless of the number of the atomic interhelical contacts. Helices involved in multiple interhelical interactions may have several unique or overlapping helical faces. We computed the best alignment to a heptad repeat (positions *a*, *d*, *e *and *g*) for all residues in every helical face.

### Data set for prediction of TM helix orientation

We collected 18 membrane proteins that have at least 10 homologous sequences for generating a good multiple sequence alignment. These proteins are listed in Table 1. The homologous sequences were found in the NR database using BLAST and PSI-BLAST searches. In most cases, we took the conservative approach and included only complete sequences with the same annotation as the query protein and with sequence identity between 35%–89% for multiple sequence alignments with ClustalW. Each multiple sequence alignment profile was edited with Pfaat multiple alignment viewer [54] to produce separate blocks of sequences with no gaps for each TM helix.

### LIPS scoring function

We use Shannon entropy *H*(*i*) to measure the level of conservation at each position *i *of the TM helical sequence as described by Larson and Davidson [25]:

H(i)=−∑rpi(r)ln⁡pi(r)     (1)
 MathType@MTEF@5@5@+=feaafiart1ev1aaatCvAUfKttLearuWrP9MDH5MBPbIqV92AaeXatLxBI9gBaebbnrfifHhDYfgasaacH8akY=wiFfYdH8Gipec8Eeeu0xXdbba9frFj0=OqFfea0dXdd9vqai=hGuQ8kuc9pgc9s8qqaq=dirpe0xb9q8qiLsFr0=vr0=vr0dc8meaabaqaciaacaGaaeqabaqabeGadaaakeaacqWGibascqGGOaakcqWGPbqAcqGGPaqkcqGH9aqpcqGHsisldaaeqbqaaiabdchaWnaaBaaaleaacqWGPbqAaeqaaOGaeiikaGIaemOCaiNaeiykaKIagiiBaWMaeiOBa4MaemiCaa3aaSbaaSqaaiabdMgaPbqabaGccqGGOaakcqWGYbGCcqGGPaqkaSqaaiabdkhaYbqab0GaeyyeIuoakiaaxMaacaWLjaWaaeWaaeaacqaIXaqmaiaawIcacaGLPaaaaaa@4914@

where *p*_*i*_(*r*) is the probability of amino acid type *r *in position *i *of the multiple sequence alignment of the TM helix. For convenience, we choose the following modification:

*E*(*i*) = *e*^*H*(*i*) ^    (2)

We calculate the average entropy and the average lipophilicity for each helical face using TMLIP2-like scale [24], which computed as regular TMLIP2 scale but without the protein that has been tested. The LIPS score *S*_*f *_for every helical face *f *is calculated as follows:

*S*_*f *_= E¯f
 MathType@MTEF@5@5@+=feaafiart1ev1aaatCvAUfKttLearuWrP9MDH5MBPbIqV92AaeXatLxBI9gBaebbnrfifHhDYfgasaacH8akY=wiFfYdH8Gipec8Eeeu0xXdbba9frFj0=OqFfea0dXdd9vqai=hGuQ8kuc9pgc9s8qqaq=dirpe0xb9q8qiLsFr0=vr0=vr0dc8meaabaqaciaacaGaaeqabaqabeGadaaakeaacuWGfbqrgaqeamaaBaaaleaacqWGMbGzaeqaaaaa@2F58@L¯f
 MathType@MTEF@5@5@+=feaafiart1ev1aaatCvAUfKttLearuWrP9MDH5MBPbIqV92AaeXatLxBI9gBaebbnrfifHhDYfgasaacH8akY=wiFfYdH8Gipec8Eeeu0xXdbba9frFj0=OqFfea0dXdd9vqai=hGuQ8kuc9pgc9s8qqaq=dirpe0xb9q8qiLsFr0=vr0=vr0dc8meaabaqaciaacaGaaeqabaqabeGadaaakeaacuWGmbatgaqeamaaBaaaleaacqWGMbGzaeqaaaaa@2F66@     (3)

where E¯f
 MathType@MTEF@5@5@+=feaafiart1ev1aaatCvAUfKttLearuWrP9MDH5MBPbIqV92AaeXatLxBI9gBaebbnrfifHhDYfgasaacH8akY=wiFfYdH8Gipec8Eeeu0xXdbba9frFj0=OqFfea0dXdd9vqai=hGuQ8kuc9pgc9s8qqaq=dirpe0xb9q8qiLsFr0=vr0=vr0dc8meaabaqaciaacaGaaeqabaqabeGadaaakeaacuWGfbqrgaqeamaaBaaaleaacqWGMbGzaeqaaaaa@2F58@ and L¯f
 MathType@MTEF@5@5@+=feaafiart1ev1aaatCvAUfKttLearuWrP9MDH5MBPbIqV92AaeXatLxBI9gBaebbnrfifHhDYfgasaacH8akY=wiFfYdH8Gipec8Eeeu0xXdbba9frFj0=OqFfea0dXdd9vqai=hGuQ8kuc9pgc9s8qqaq=dirpe0xb9q8qiLsFr0=vr0=vr0dc8meaabaqaciaacaGaaeqabaqabeGadaaakeaacuWGmbatgaqeamaaBaaaleaacqWGMbGzaeqaaaaa@2F66@ are average entropy and average lipophilicity of the helical face, respectively.

We use the VOLBL method [55] to compute lipid-accessible residues and their surface area as described previously [24].

Sequence logos were built using weblogo generator [56].

## Authors' contributions

Both authors equally contributed to this work.

## Supplementary Material

Additional File 1 **Comparison of the failed predicted *vs*. correct buried faces**. The correct faces have significant overlap with the predicted faces and differ only by one residue slide (rotation) of the helical wheel in 28 helices, and by two residue slides in 18 helices. Click here for file

Additional File 2 **Multiple sequence alignments for PetG, PetL, PetM, and PetN TM helices of cytochrome b**_6_**f**. The first sequence in every sequence alignment is from 1Q90 structure, while the second sequence is from 1VF5 structure. Helix boundaries are based on 1Q90 structure assignment. Click here for file
