# Supplementary material for: Prediction of transmembrane helix orientation in polytopic membrane proteins
Source: BMC Struct Biol. 2006 Jun 22;6:13. doi: 10.1186/1472-6807-6-13 (PMC1540425; doi:10.1186/1472-6807-6-13)
Supplement: Additional File 1 — Comparison of the failed predicted vs. correct buried faces. The correct faces have significant overlap with the predicted faces and differ only by one residue slide (rotation) of the helical wheel in 28 helices, and by two residue slides in 18 helices. [file 1472-6807-6-13-S1.doc]

Supplementary Table 1 – Comparison of the failed predicted *vs.* correct buried faces.

The correct faces have significant overlap with the predicted faces and differ only by one residue slide (rotation) of the helical wheel in 28 helices, and by two residue slides in 18 helices.

| **Protein** | **Helix^1^** | **Predicted**  **Face** | **Correct**  **Face** | **Rotation^2^** |
| --- | --- | --- | --- | --- |
| 1C3W | 4 | 6 | 3 | 1 |
| **1EUL** | 1  8 | 4  3 | 3  6 | 2  1 |
| **1FX8** | 5 | 1 | 2 | 2 |
| **1IWG** | 2  5  7  8 | 6  4  4  3 | 3  1  3  0 | 1  1  2  1 |
| **1KPL** | 11 | 5 | 2 | 1 |
| **1KQF** | 1  2 | 2  3 | 6  2 | 1  2 |
| **1M3X** | 9  10  11 | 4  5  0 | 3  6  3 | 2  2  1 |
| **1NEK** | 2  3 | 5  2 | 2  1 | 1  2 |
| **1OCR** | 5  7  8  9  13  18  19  21  22  23  24  26  28 | 4  0  4  2  4  5  0  0  0  0  3  4  1 | 5  1  5  6  1  2  3  3  3  3  6  1  4 | 2  2  2  1  1  1  1  1  1  1  1  1  1 |
| **1OKC** | 2  4  6 | 5  1  4 | 1  2  1 | 1  2  1 |
| **1PV6** | 1  2  3  4  5  7 | 6  1  6  5  1  3 | 2  4  3  3  4  2 | 1  1  1  3  1  2 |
| **1PW4** | 10 | 1 | 0 | 2 |
| **1Q90** | 1 | 1 | 2 | 2 |
| **1RH5** | 4  5  6  7  8  11 | 3  0  3  5  6  5 | 2  5  6  4  3  1 | 2  3  1  2  1  1 |
| **1ZCD** | 9  10 | 0  1 | 1  2 | 2  2 |

^1^ TM helices are consequently numbered as they appear in the X-ray structures.

^2^ Rotation in terms of the number of residues in a heptad repeat between correct face and predicted face.
