# Supplementary material for: Prediction of transmembrane helix orientation in polytopic membrane proteins
Source: BMC Struct Biol. 2006 Jun 22;6:13. doi: 10.1186/1472-6807-6-13 (PMC1540425; doi:10.1186/1472-6807-6-13)
Supplement: Additional File 2 — Multiple sequence alignments for PetG, PetL, PetM, and PetN TM helices of cytochrome b6f. The first sequence in every sequence alignment is from 1Q90 structure, while the second sequence is from 1VF5 structure. Helix boundaries are based on 1Q90 structure assignment. [file 1472-6807-6-13-S2.doc]

**Additional File 2 - Multiple sequence alignments for PetG, PetL, PetM, and PetN TM helices of cytochrome b_6_f**

The first sequence in every sequence alignment is from 1Q90 structure, while the second sequence is from 1VF5 structure. Helix boundaries are based on 1Q90 structure assignment.

>PetG (LEU G 5 – LEU G 27)

LLCGIVLGLVPVTIAGLFVTAYL

LLDGLVLGLVFATLGGLFYAAYQ

LLSGIVLGLIVVTLAGLFYAAYK

LLSGVVLGLILVTLSGLFFAAYQ

LLLGIVLGLIPVTLAGLFVAAYL

LLCGIVLGLIPVTLAGLFFAAYQ

LLSGIVLGLVPITITGLLVTAYL

LLSGIVLGLIPITLAGLFVTAYL

FLFGIVLGLIPITLTGLFVTAYL

LLSGIVLGLIPITILGLLMAAYF

LLTGIVLGSIFITLLGLLAAAKL

LLSGIILGLIPITICGLFFTAYL

LLSGIVLGLVPVTITGLFVAAYL

LLSGIVLGMITVSAFGLFVAAFL

LLSGIILGLIPVTLSGLLVAAYL

LLSGIVLGLIPVTLFGLLVAAYL

LLCGIVLGLIPITLAGLFMAAYL

LLCGIVLGLVPITLLGLFVSAWN

LLCGIVLGLIPVTLLGLFVAAWN

LLCGIVLGLIPVTLLGLFVDAWN

ILLGMVLGFVPVTIAGLLVAAWL

>PetL (MET L 1 – LEU L 26)

MLTITSYVGLLIGALVFTLGIYLGLL

ILGAVFYIVFIALFFGIAVGIIFAIK

LLLMVEYLVILSGMFGLALACFFGLR

MSALIGYILLMTLMFSLAAGLYFGLR

MSLIIGYIILLACAFGLAAGLYFGLS

MLAIVAYIGFLALFTGIAAGLLFGLR

MSLFIGYIIFLVAFFGLATGLFLGLK

LMGILFYLVFVGAGLSAAFLIQKALK

MFTLTSYFGFLLAALTITFVLFIGLN

MLTLTSYFGFLLAALTITSALFIGLS

MFTSLSYFAFLMLALTFTLALFVGLN

>PetM (GLU M 64 – LEU M 93)

EFIAGTALTMVGMTLVGLAIGFVLLRVESL

EEMLYAALLSFGLIFVGWGLGVLLLKIQGA

GELLNAALLSFGLIFVGWALGALLLKIQGA

KEIVESAILSSVLVLVGLAVGFLLLKVQGE

GEIFGTAFLFIVLVPVGLALGAFLLKVQGV

SMLANGAFIMIGLTLLGLAWGFVIIKLQGS

SEIFNAAVTCIFMTLFGLSLGFALLKVQGE

SMMFNGAVVLMVLVLFGLAWGFLILKIQGG

AEIVTAAVTCIFMVLFGLSLGFALLKVQGE

NEFIASASISFIITLIGLTLGFALLKLQGE

KQIFNTAVICFTLTLIGLSLGFVLLKIQGD

EEIFNTAVITFTLVLVGLGAGYLLLRLTPD

GFIAGTAFTMIGITLLGLAVGFVLLRVEAA

GEIVQIAATLNGLTLLGVAVGFVLLRIEAF

GEIFKIAAIMNALTLVGVAVGFVLLRIETS

GEIFRIAVIMNGLVLVGVAVGFVLLRVEAA

AEIFRIAAVMNGLTLVGVAIGFVLLRIEAT

>PetN (ILE N 72 – ARG N 95)

IVQIGWAATCVMFSFSLSLVVWGR

IDVLGWVALLVVFTWSIAMVVWGR

IISLGWAGLMTMFTFSLALVVWAR

LLSLGWSSLMVMFSFSLALVVWGR

LITITWASVMVAFTFSLSLVVWGR

ILSLGWSALMVVFTFSLALVVWGR

IVSLAWAALMVVLHFSLSLVVWGR

IVSLTWAALMVVFTFSLSLVVWGR

IVGIAWAALMVVFTFSLSLVVWGR

IINIAWAALMVIFTFSLSLVVWGR

IVNIAWGALMVMFTFSLSLVVWGR

IVNIAWAALMVVSTSSLSPVAWGR

IVNIAWAALMVVSTFSLTLVVWGR

TVSIAWAALMVIFTFSISLVVWGR

SVTIAWAALMAISTFSLSLVVRGR

IVSAGWAFLMVSFTFSLSLVVWGR

LVDLTWACLMVSFTVSLALVVWAR

LISITWGCLMATFTASLALVIWAR

IISLGWVFLMVFFSFSLSLVVWAR

ILSLGWAALMASFTFSLSLVVWGR

LFTLAWASLAAVFSFSIAMVVWGR

IFTLGWASLAAIFTFSIAMVVWGR

LFTFAWASLAAIFTFSIAMVVWGR

IFQIGWAALAAIFTFSIAMVVWGR

LLTFGWAALLAVFTFSLAMVVWGR

ILSLGWAALMAMFTFSIAMVVWGR

IISLGWGSLLAIFSFSIALVVWGR

DPTVGWIALLAFFVVSIALVVWGR

ILTLGWVSLLVVFTWSIAMVVWGR

ILTLGWVSVLVLFTWSISMVVWGR

IITLGWVGVLSVFTLSIAFVVWGR
